# Variance in Landscape Connectivity Shifts Microbial Population Scaling

**DOI:** 10.3389/fmicb.2022.831790

**Published:** 2022-04-01

**Authors:** Miles T. Wetherington, Krisztina Nagy, László Dér, Janneke Noorlag, Peter Galajda, Juan E. Keymer

**Affiliations:** ^1^Department of Ecology, School of Biological Sciences, P. Catholic University of Chile, Santiago, Chile; ^2^Institute of Biophysics, Biological Research Centre, Szeged, Hungary; ^3^School of Applied and Engineering Physics, Cornell University, Ithaca, NY, United States; ^4^Department of Natural Sciences and Technology, University of Aysén, Coyhaique, Chile

**Keywords:** landscape ecology, metapopulations, spatial microbial ecology, Taylor's Law, scaling laws, microfluidics

## Abstract

Understanding mechanisms shaping distributions and interactions of soil microbes is essential for determining their impact on large scale ecosystem services, such as carbon sequestration, climate regulation, waste decomposition, and nutrient cycling. As the functional unit of soil ecosystems, we focus our attention on the spatial structure of soil macroaggregates. Emulating this complex physico-chemical environment as a patchy habitat landscape we investigate *on-chip* the effect of changing the connectivity features of this landscape as *Escherichia coli* forms a metapopulation. We analyze the distributions of *E. coli* occupancy using Taylor's law, an empirical law in ecology which asserts that the fluctuations in populations is a power law function of the mean. We provide experimental evidence that bacterial metapopulations in patchy habitat landscapes on microchips follow this law. Furthermore, we find that increased variance of patch-corridor connectivity leads to a qualitative transition in the fluctuation scaling. We discuss these results in the context of the spatial ecology of microbes in soil.

## Introduction

Soil microbial inhabitants provide numerous ecosystem services that are fundamental for the persistence of multicellular life on earth (Smith et al., 2015). Despite the central role these communities play in the flow of the nutrient cycles, primary production and climate regulation underpinning earths biosphere, we still know very little about the processes shaping their dispersal, colonization dynamics, and overall spatial distributions (Raynaud and Nunan, 2014). This is due in part to the complex physico-chemical spatial structure generated by soil (Ettema and Wardle, 2002). Soil aggregate structure plays a crucial role in community and organic matter dynamics (Six et al., 2004; Young and Crawford, 2004; Bailey et al., 2013), as well as a feedback between the two (Ebrahimi and Or, 2016). The coalescence of soil microaggregates (<250μm) giving rise to macroaggregates (0.25–2 mm) generates an enclosed patchy habitat landscape (Jastrow et al., 1996) which serves as a discrete functional unit of the soil microbial ecosystem (Wilpiszeski et al., 2019).

Developing a statistical mechanics characterizing patterns of microbial species abundance and distribution in such habitat landscapes is a fundamental goal of ecology (Brown et al., 1995; Xu et al., 2020). One such pattern is Taylor's Law (TL) which states that the variance in the occupancy of a (meta-) population $\sigma_{\phi }^{2}$, is related to the mean 〈ϕ〉, by a power law (Taylor, 1961):


(1) $$\begin{array}{r} \sigma_{\phi }^{2}=c{\langle \phi \rangle }^{\alpha } \end{array}$$

Often considering population ensembles in space or time, this statistical emergent property of populations was first shown empirically by L. R. Taylor when he compiled census data of several organisms, including macro-zooplankton, worms, insects, mites, and fish (Taylor, 1961). Since this landmark paper, TL as a statistical phenomena has been observed far beyond the scope of ecology, present in cell biology (Azevedo and Leroi, 2001), linguistics (Tanaka-Ishii and Kobayashi, 2018), social science (Xu and Cohen, 2019), and even number theory (Cohen, 2016) in mathematics (see Eisler et al., 2008 for a review).

The case for TL Universality in ecological populations has been strengthened, more recently having been confirmed in metagenomic studies of microbes in the human gut (Ma, 2015), hot spring (Li and Ma, 2019), and several other microbiomes (Grilli, 2020). While the phenomena of power law scaling appears to be a universal property of populations, the slope (α) varies: In his own words, Taylor considered α to be the “index of aggregation” ranging from near regular dispersion α → 0, to random (Poisson) α = 1 to aggregated α = 2 (Gamma) and beyond α → ∞. Given the cornucopia of slopes these results produced, Taylor considered α to be a characteristic feature of the organism in question.

Beyond simply the verification of its existence, TL has provided insight into aspects of spatial ecology, including a connection with the Moran effect (Reuman et al., 2017), which describes the synchronization of dispersed populations within a landscape that are environmentally correlated (Moran, 1953). While underlying mechanisms for the origins of differing α's are still up for debate (Fronczak and Fronczak, 2010; Kendal and Jørgensen, 2011; Cohen and Xu, 2015), one thing is certain; α varies considerably from organism to organism and from landscape to landscape.

Despite the recognition that landscape structure impacts metapopulation dynamics (Wiens, 1997), as of yet, no connection has been made explicitly between the physical properties of microbial ecological landscapes and their impact on the slope (α) of the metapopulation. The aforementioned metagenomic studies, while providing a significant first step toward connecting TL to microbiome types, lack the capacity to connect to spatial characteristics of their abiotic surroundings at the scale of micro and macro soil aggregates. Performing such an investigation requires precise control of physical properties defining the landscape in question.

**Figure 1:**
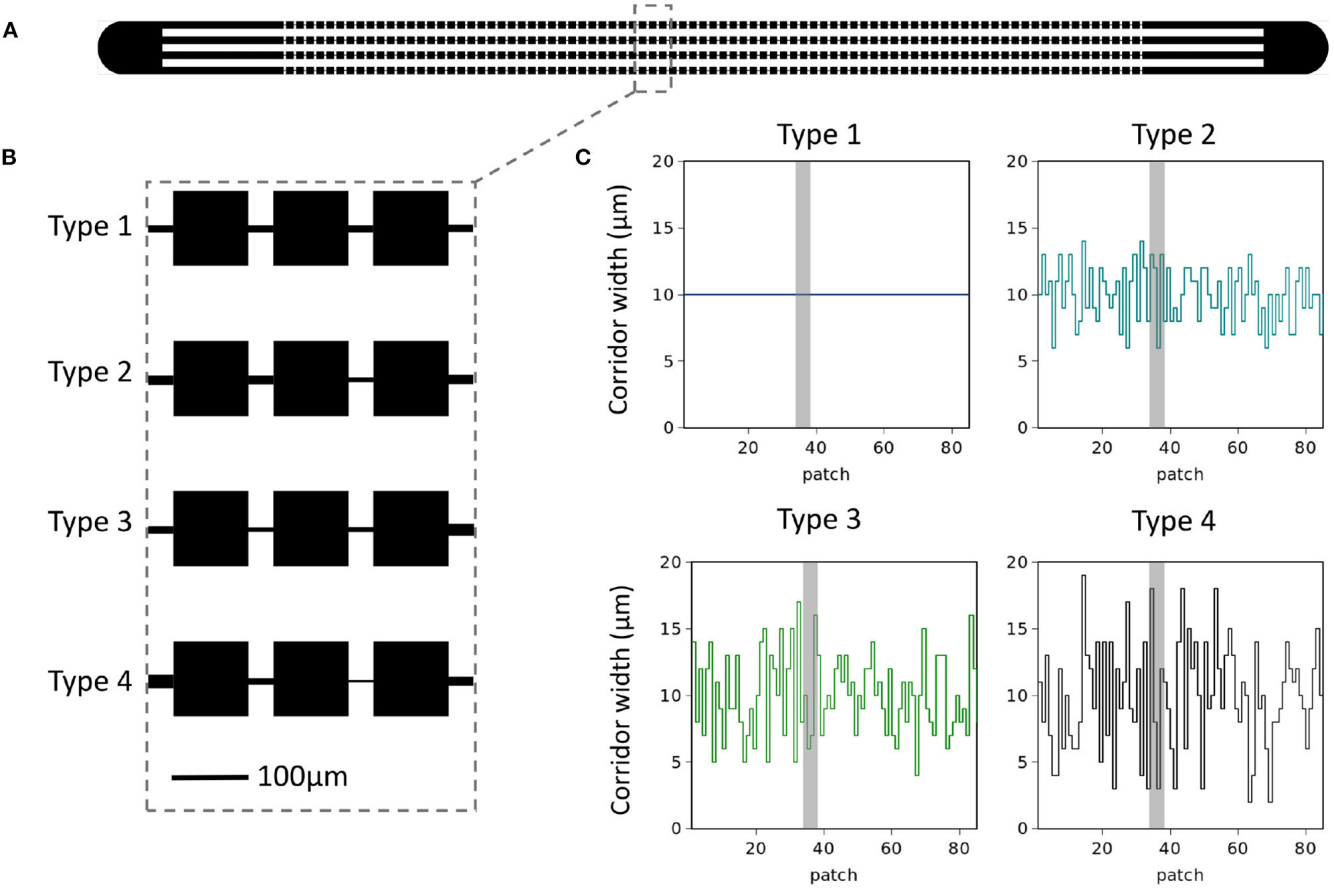
Microfluidics device. **(A)** A sketch of the microfluidics device with single inlets on each side leading into four parallel habitat landscapes. **(B)** Zoom-in view of patch-corridor structure in each of the four parallel landscapes. For type 1 corridor width is kept constant at 10μm. Types 2–4 the average width is also 10 μm with variance around the mean increasing from type 1 to 4 as σ^2^ = 0, 4, 9, 16 μm^2^. This can be appreciated in **(C)** where we show the pattern of corridor widths used for each landscape type. Grey shading indicates location of zoom-in.

In this work, we show results from microfluidic experiments (see Materials and Methods) using a single species of bacteria, *Escherichia coli*, which is known to form a metapopulation in such micro-habitat patch landscapes (Figure 1A) and therefore suitable for studying in the context of TL (Keymer et al., 2006). As a first step in connecting landscape microbial ecology to Taylor's Law, we aim to answer the following questions; (i) does the long-term metapopulation occupancy structure display a discernible α? And, if so, (ii) is α landscape structure dependent?

## Results

We compared *E. coli* metapopulation distributions in different landscape types (Figure 1). In order to achieve this, we studied the distribution of *E. coli* occupancy in microfluidic devices which consist of microscopic chambers (patches) connected by narrow channels (ecological corridors). Here, the only difference between landscape types was the within-landscape corridor width variance (see Figure 1C).

Starting from landscape type 1, which acts as our idealized patchy habitat landscape, we steadily begin to increase variance in our landscapes, while maintaining a landscape average corridor width of 10 μm. From Figure 1B we see a zoom-in depicting how the fluctuations around 10 μm arise for each of the landscape types. Higher moments and spatial auto-correlations were not considered and all experiments (*n* = 30 replicates) were conducted with the exact landscape structures depicted in Figure 1C. After a relaxation time of 48 h after inoculation, images were captured from which patch occupancy (the percentage of the patch occupied by *E. coli*) can be deduced (see Materials and Methods). For the remainder of this work we discuss this “steady-state” distribution of *E. coli* metapopulations in the four varying landscapes.

**Figure 2:**
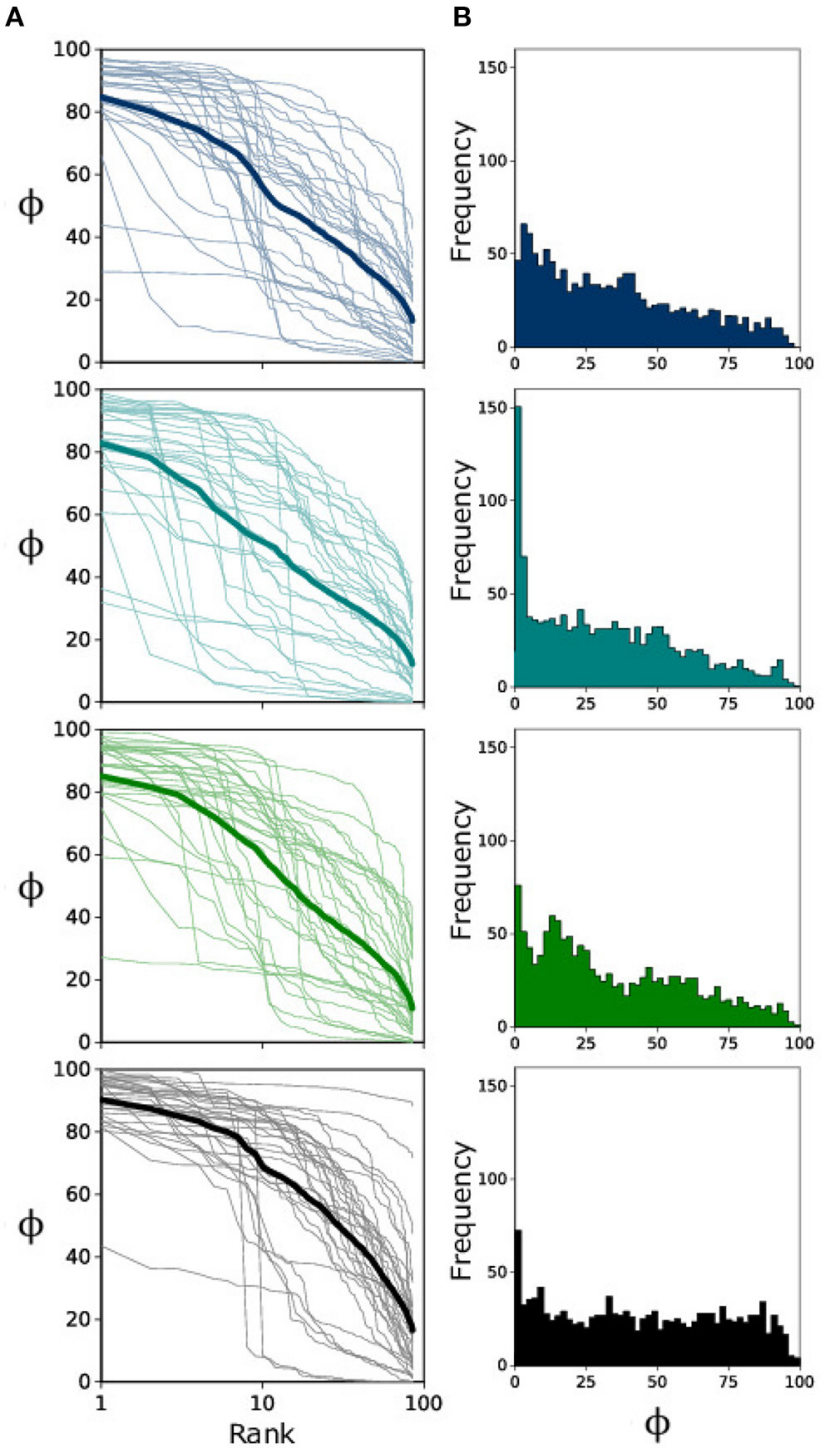
**(A)** Semi-log plot of Rank-Size Distribution for all landscape replicates (*n* = 30) in light color and the ensemble average in bold. Occupancy ranges from 0 to 100%. **(B)** Occupancy frequency histogram for individual patches (*n* = 2,550).

In Figure 2A, we consider the Rank-Size Distribution (RSD) for each landscape type; this shows us how occupancy changes from most occupied patch (Rank 1) to least occupied patch (Rank 85) for each individual experiment (thin transparent lines) and for the cumulative average (bold line) over all (*n* = 30) replicates. From this figure it is clear that there is a great deal of variation in how the metapopulation becomes distributed. Surprisingly, if we consider the frequency of occupancy values for all patches (Figure 2B) we see that, unlike landscape types 1–3 which exhibit the expected decay in frequency from low-to-high occupancy, landscape type 4 seems to render a relatively invariant frequency for occupancy values.

Guided by these results we take a novel approach to computing Taylor's Law using the RSD data to generate means and variances which we will compare to a more traditional spatial TL. Our concern is with understanding whether distribution differs between landscape types. Contrary to the spatial TL, where mean and variance is computed using the same patch number for all replicates (*n* = 30), RSD TL mean and variance is computed using the same patch rank. For RSD TL only mean patch occupancy rankings <50% are used to compute power-law slopes (see Materials and Methods). The RSD TL approach allows us to take the spatial mapping of the metapopulation out of the equation which is in focus using the spatial TL, and instead spotlight the distributional response to the varying landscape types by *E. coli* reflected in the RSD results.

**Figure 3:**
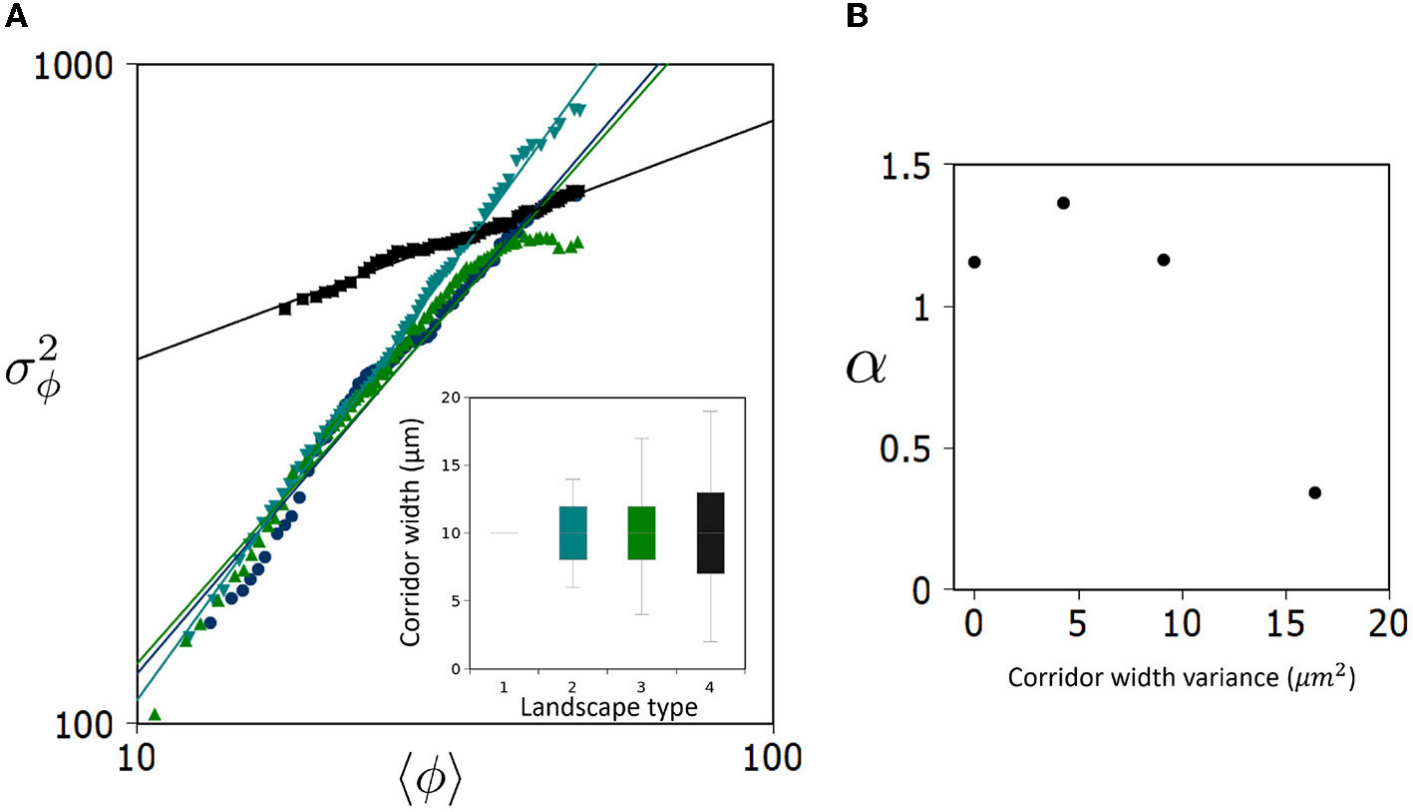
Landscape dependency of Taylor's Law in a bacterial metapopulation. **(A)** Log-log plot showing the relationship between the average occupancy of a patch, 〈ϕ〉, and its variance, $\sigma_{\phi }^{2}$, over (*n* = 30) replicates and across all patches (85) generating the unique Taylor's Law (TL) for each landscape type. Inset displays a box plot of corridor widths around the mean (10 μm). Four variances were used in this study, (σ^2^ = 0, 4, 9, 16μ*m*^2^). **(B)** The slope of TL, α, is plotted against the variance of corridor width (randomness) defining landscape types.

In Figure 3A, we see that for landscape types 1–3, α values fall between 1.1 and 1.4 which is within the range commonly found in the literature (Eisler et al., 2008). Strikingly, landscape type 4 does not follow suit, instead registering a value of α_4_≈0.34 (Figure 3). These results suggest that beyond some critical level of corridor variance, in this case ($9\mu m^{2}<\sigma_{crit}^{2}<16\mu m^{2}$), a qualitative change occurs in the long-term distribution of *E. coli* metapopulations.

When interpreting these results, it is useful to consider two regimes of the metapopulation occupancy: For relatively low density sub-populations occupying patches in the metapopulation, where landscape occupancy 〈ϕ〉 < <50%, type 4 patches have a significantly higher fluctuation around the mean than equivalent occupancy patches in landscape types 1–3 (Figure 3A). To this effect, the normalization constant (*c*) would be characterized as follows: *c*_4_>*c*_1−3_. However, given the scaling behavior observed (α_4_ < α_1−3_), this disparity diminishes as we consider more concentrated patches in the metapopulation 〈ϕ〉 → 50% where the mean occupancy of concentrated patches in all landscape types tends to fluctuate similarly.

A key takeaway from our results is that we see the fluctuation scaling of bacterial metapopulations change with landscape modifications. For landscape 4 (which has the largest amount of corridor variance) we see that, when considering the power-law scaling of Rank-Size Distributions ({flreffig3Figure 3A), the less occupied patches tend to have much higher variance than their counterparts in landscapes 1–3. On the contrary, higher occupancy averages behave similar to the other landscapes, thus rendering a shallower power-law slope and lower value for α_4_. This uniform distribution is reflected well in the lowest panel of Figure 2B which does not display as pronounced a decay in occupancy frequency. These results suggest a qualitative transition in the spatial distribution of *E. coli* when establishing metapopulations in landscapes with large variance in ecological corridors.

Particularly, we observe an abrupt transition in the Taylor's Law slope for landscape 4, as we see a relatively consistent value for α below $\sigma_{crit}^{2}$, i.e., landscape types 1–3. This “basal” α value while differing quantitatively between the three landscape types remains qualitatively the same, lying between a random/Poisson distribution (α = 1) and aggregated/gamma distribution (α = 2). Counter-intuitively, landscape randomness (above $\sigma_{crit}^{2}$) induces a qualitative change driving the overall scaling of variance in the metapopulation to be more uniformly distributed (α <1). However, when considering only fluctuations for low occupancy ensembles, we see a dramatic increase in randomness in metapopulation distribution.

## Discussion

It remains to be seen exactly how these metapopulations arrive at this alternative statistical equilibrium. It is well-known that *E. coli* collectively migrate into such landscapes as traveling chemotactic waves (Saragosti et al., 2011; Van Vliet et al., 2014). Therefore, following the range-expansion dynamics of pioneering population waves is a logical next step in answering this question. One possible explanation for these sharp transitions in metapopulation distribution is due to self-driven jamming by bacterial waves as they traverse the landscape. This spatial ecology scenario with planktonic, free swimming cell populations may parallel the dynamics of traffic jams (Nagatani, 2002). While speculative—as this research is ongoing—it would be an interesting contribution to the current field of “jamming” in microbial systems which has mainly focused on how mechanical pressure imposed by cell division in confined spaces effects single cell physiology (Delarue et al., 2016).

Previous investigations by Van Vliet et al. (2014) using the same strain and similar microfluidics devices, also designed with shared inlets leading to multiple landscapes, yet without variance in ecological corridor width, showed strikingly similar patterns of colonization and occupancy. In fact, van Vliet et al. found more similar occupancy patterns for experiments performed with shared inlets and identical colonies than those performed on independent inlet devices and different colonies. Taking this into account and the extensive number of replicate colonies performed for this investigation suggests that our results are a property of the spatial biology at play within the habitat landscapes and not a methodological artifact, nor a stochastic feature of under-sampling.

Our results reflect the conclusions of Reuman et al. (2017) suggesting α is not only a trait of a species—originally asserted by Taylor—but likely a tunable characteristic which responds to external factors as in the case of the Moran effect, *sensu*, or in this case—landscape properties. One question which remains is if there exists a “basal” α for populations that is only perturbed by exceptional external disturbances within the landscape or strong trophic or competitive interactions. That we should see such shifts in the scaling exponent α driven by external factors does not diminish the utility of TL, rather it refines our understanding and in fact marks development and progress as seen in other ecological scaling laws (Marquet et al., 2005).

In this light, pursuit of these questions will aid in finding connections between TL and other ecological scaling laws which is an exciting and ongoing effort (Zaoli et al., 2017). It is clear, extensions to the microbial world are in order and likely to provide unexpected results. This is especially the case given the known disparity of metabolic scaling observed between prokaryotes and the more commonly studied metazoa (DeLong et al., 2010). The application of a RSD approach to Taylor's Law could be of use when dealing with metagenomic data, since fine-grained spatial structure is eliminated in sample processing. Notwithstanding, we believe controlled spatial experiments like those made accessible through the lab-on-a-chip framework will play an important role.

## Materials and Methods

###  Strains and Growth Conditions

Experiments were performed using *E. coli* (JEK1036) labeled with Green Fluorescent Protein (GFP) (Keymer et al., 2006) which is induced with Isopropyl β-D-1-thiogalactopyranoside (IPTG). Single colonies were grown from −80°C glycerol stocks on solid LB agar plates (LB Broth EZMix, Sigma-Aldrich + 1.5% Bacto Agar, MOLAR Chemicals) and subsequently inoculated in 3 ml Lysogeny Broth medium (LB Broth EZMix, Sigma-Aldrich) for 16 h ± 30 min (O/N) at 30°C, 200rpm. Overnight cultures were back-diluted 1:1,000 in 3ml LB medium with 1mM IPTG and grown to an optical density at 600nm (OD_600_) of 0.3. Cultures were then centrifuged at 350G for 10 min, after which the supernatant was removed and cells were resuspended in LB medium containing 1mM IPTG.

###  Microfluidic Device Fabrication and Preparation

Microfabricated devices used in this study consist of two inlet holes (1.2mm) on opposite sides with four parallel landscapes, each with 1-dimensional arrays of 85 habitat patches (100 × 100 × 5μm^3^) connected by corridors with constant lengths (50 μm) and depths (5 μm) but with different widths.

Devices were fabricated using soft lithography techniques (Qin et al., 2010): A silicon wafer was coated with a thin film (5 μm, height of the device) of the negative photoresist SU-8 (SU-8 2005, MicroChem) and the design of the device was written into the resist with a laser pattern generator (μPG 101, Heidelberg Instruments) to fabricate a master mold on which Polydimethylsiloxane (10:1 PDMS:curing agent, Sylgard 184, Dow Corning) was deposited to yield an elastomeric stamp that was covalently bonded to a glass cover slip by oxygen plasma activation (29.6 W, 400 mTorr, 45 s; PDC-002, Harrick Plasma) of both the PDMS and glass parts.

#### Microfluidic Experiments

Prior to inoculation, the devices were wettened with LB + 1mM IPTG. Then, 1μl culture was pipetted into one inlet hole. Once inoculated on one side, the device was sealed with fast curing PDMS (Kwik-Sil Silicone Elastomer, World Precision Instruments). A water tight wall is made around the perimeter of the sealed device on the glass slide using four 24 × 60mm coverslips which were previously “painted” up-right onto the glass-slide using fast curing PDMS. The sealed device was then submerged in Milli-Q water and placed into an incubator at 30°C for 48 h. This was performed in order to insure no drying of devices over the 48 h incubation time.

## Image Acquisition and Data Analysis

After 48 h of incubation, devices were imaged using a Nikon Eclipse Ti-E microscope equipped with 10X Nikon Plan Fluor objective, GFP fluorescence filter set (49002 Chroma Inc.), Andor Neo sCMOS camera (Andor Technology plc.), LUMEN 200 Pro metal arc lamp (Prior Scientific Ltd.) and a Prior Proscan II motorized stage (Prior Scientific Ltd.) was used for scanning. The NIS Elements Ar software (Nikon Inc.) was used for image acquisition and data processing and image analysis was carried out using ImageJ and Python. Fluorescence intensity is a poor estimation for biomass due to differences in expression among cells. To avoid this problem we use a custom script in ImageJ to convert all images to occupancy data.

### Local Occupancy Within Patches (MHPs)

After background correction is performed on each image a threshold pixel value is calculated based off the auto-fluorescence for the green color channel used. When the value is above this threshold, the pixel (0.803μm^2^) is considered occupied (value of 1), otherwise it is vacant (value of 0). Using the ROImanager in ImageJ (Abràmoff et al., 2004), a custom mask was fitted at each patch for each experiment allowing us to consider occupancy values at the patch level.

### RSD and Spatial Taylor's Law

Occupancy for a MHP spatially indexed by *i* is denoted as ϕ_*i*_, thus 0 ≤ ϕ_*i*_ ≤ 100% occupancy. Running *n* = 30 replicates, and ranking patches with rank index *k* from highest *k* = 1 to lowest *k* = 85 an ensemble average 〈ϕ〉_*k*_ generated from all *n* = 30 replicates can be computed for each rank *k* (bold line in Figure 3A). Next, a variance is computed for each rank *k*, finally, ∀〈ϕ〉_*k*_ ≤ 50% we generate a best fit linear regression for our data in log-log form. This can be written as $\sigma_{\phi k}^{2}=c{\langle \phi \rangle }_{k}^{\alpha }$ with the condition ∀〈ϕ〉_*k*_ ≤ 50%.

For values 〈ϕ〉_*k*_>50% variance begins to decrease. There are reasons related to the binary entropy function. First consider pixel occupancy as our random variable *X* that can only take two mutually exclusive values; vacant (0) or occupied (1). If a patch is 50% occupied it's entropy [*H*(*X*)] is at its maximum value; assuming a unbiased coin flip determines the outcome of individual pixel values, probability *p* = 0.5 uncertainty is maximized. Likewise, mean patch values of 50% occupancy maximizes variance. Since in the RSD TL we are averaging over "like" patch types, that is, all patches in the 30 replicates with rank 85,...,1. For all patches rank 1 we can expect higher values than rank 2,...,85. In doing so we have narrowed the range of expected values and thus decreased variance. To illustrate this, all data is shown in Supplementary Figure 1.

Next we considered the spatial TL. For this, an average, 〈ϕ〉, and variance, $\sigma_{\phi }^{2}$, is calculated for each patch *i* within each of the four landscape types, rendering 85x$\left[\langle \phi \rangle ,\sigma_{\phi }^{2}\right]$ data points. The best fit linear regression of log 〈ϕ〉 and log $\sigma_{\phi }^{2}$ is shown in Supplementary Figure 2. This version of TL can be written as $\sigma_{\phi i}^{2}=c{\langle \phi \rangle }_{i}^{\alpha_{s}}$ where *i* = 1, ..., 85 patch indices.

Finally, we compared slopes obtained from the RSD TL to those using the spatial TL for each of the four landscapes in Supplementary Figure 3. We find qualitative agreement between the two techniques, and particularly, for landscape type 4 which exhibits sub-Poisson distribution regardless and in contrast to the other three landscape types.

## Data Availability Statement

The original contributions presented in the study are included in the article/Supplementary Material, further inquiries can be directed to the corresponding author/s.

## Author Contributions

MW participated in conceiving the study and in its design, performed the experiments and data analysis, and participated in drafting the manuscript. LD contributed to data analysis. KN participated in performing the experiments. JN participated in the design of the study and drafting the manuscript. PG participated in conceiving the study, in its design, in drafting the manuscript, and helped perform experiments. JK participated in conceiving the study, participated in its design, and in drafting the manuscript. All authors contributed to the article and approved the submitted version.

## Funding

JK, MW, and JN acknowledge financial support from CONICYT FONDECYT grants 1150430 and 1191893. JK would like to thank ANID—Millennium Science Initiative Program—NCN19_170 for their support. MW gratefully acknowledges support from the James S. McDonnell Foundation Postdoctoral Fellowship award (https://doi.org/10.37717/2020-1543) as well as CONICYT-PFCHA/Doctorado Nacional/2019-21191687. KN and PG acknowledge the support of the Hungarian National Research, Development and Innovation Office under grant numbers PD 124889 and K 116516. KN was supported by the János Bolyai Research Scholarship of the Hungarian Academy of Sciences (BO/00463/18/8). Furthermore, this work was supported by the Hungarian Government and the European Regional Development Fund under the grant numbers GINOP-2.3.2-15-2016-00001, GINOP-2.3.2-15-2016-00026, and GINOP-2.3.2-15-2016-00037.

## Conflict of Interest

The authors declare that the research was conducted in the absence of any commercial or financial relationships that could be construed as a potential conflict of interest.

## Publisher's Note

All claims expressed in this article are solely those of the authors and do not necessarily represent those of their affiliated organizations, or those of the publisher, the editors and the reviewers. Any product that may be evaluated in this article, or claim that may be made by its manufacturer, is not guaranteed or endorsed by the publisher.

## Supplementary Material

Supplementary Figure 1 Full data of RSD TL including (ϕ) > 50% occupancy. See Materials and Methods.

Supplementary Figure 2 Spatial TL values α_*s*_ for landscape types 1−4 from left to right and top to bottom are 1.228, 1.558, 1.085, 0.646. See Materials and Methods for details.

Supplementary Figure 3 Spatial and RSD TL slopes. X-axis are values shown in main text for the RSD TL. Y-axis are slopes α_*s*_ computed for same data using spatial TL method. Black line with slope = 1 to guide the eye.
