# Supplementary figures and images for: Variance in Landscape Connectivity Shifts Microbial Population Scaling
Source: Front Microbiol. 2022 Apr 1;13:831790. doi: 10.3389/fmicb.2022.831790 (PMC9020879; doi:10.3389/fmicb.2022.831790)

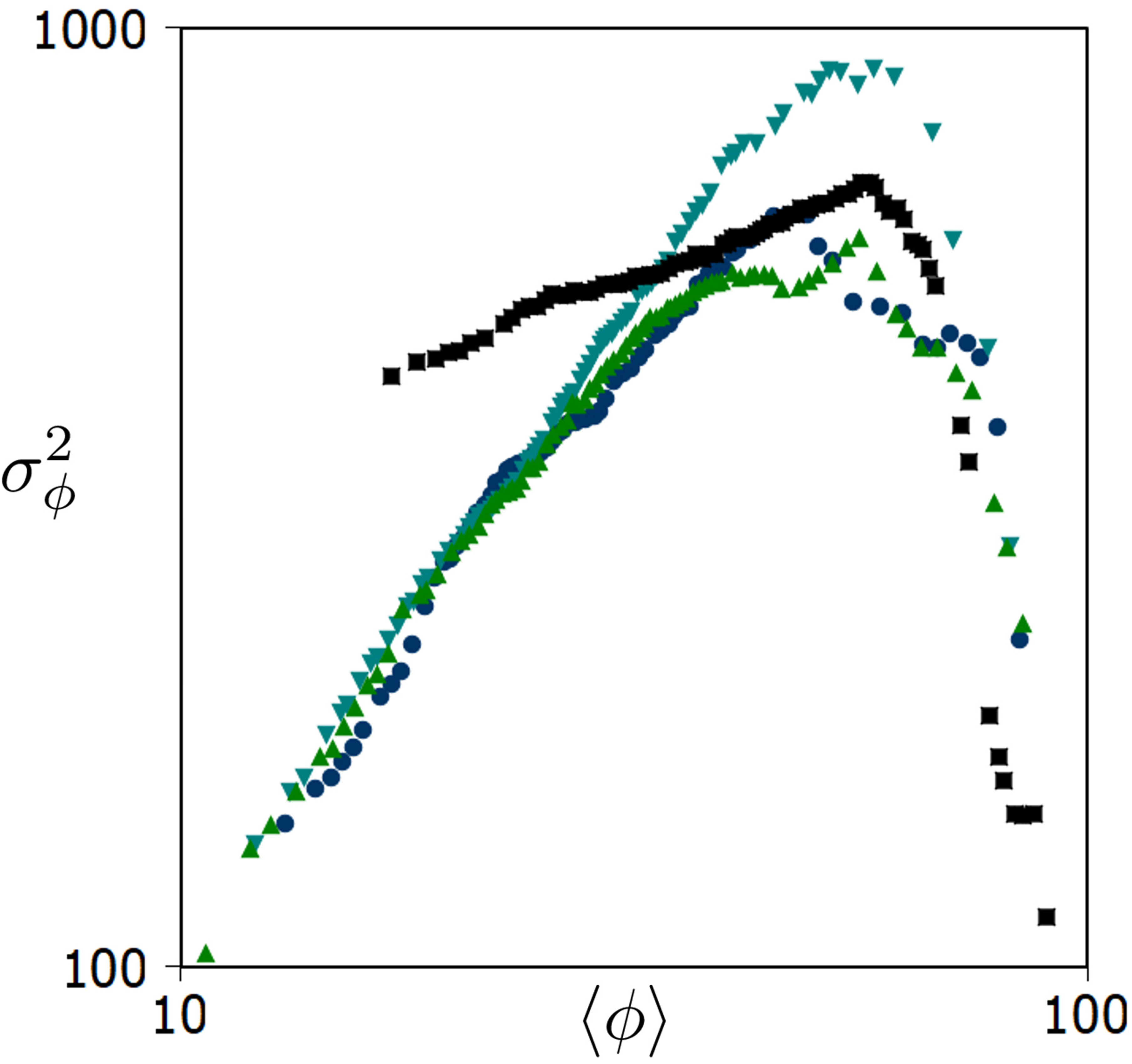

Supplement: Supplementary Figure 1 — Full data of RSD TL including (ϕ) > 50% occupancy. See Materials and Methods. [file Image_1.jpg]

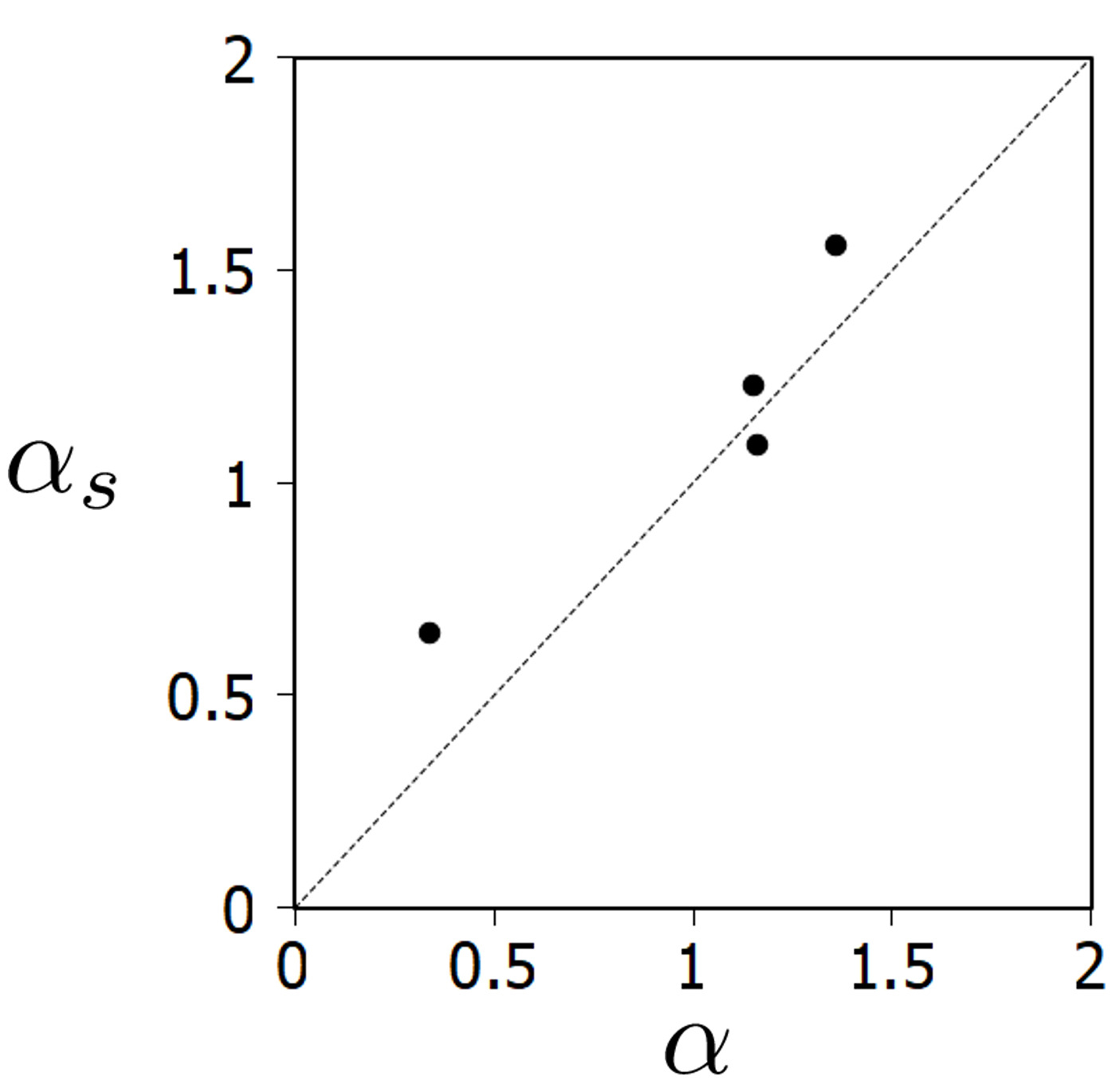

Supplement: Supplementary Figure 3 — Spatial and RSD TL slopes. X-axis are values shown in main text for the RSD TL. Y-axis are slopes αs computed for same data using spatial TL method. Black line with slope = 1 to guide the eye. [file Image_3.jpg]
